# Marine Microorganisms: perspectives for getting involved in cellulosic ethanol

**DOI:** 10.1186/2191-0855-2-46

**Published:** 2012-08-29

**Authors:** Pablo Intriago

**Affiliations:** 1 Empagran, Km 19 ½ via a la Costa, Guayaquil, Ecuador; 2 South Florida Farming Corp. 3232 Coral Way, Suite 1201, Miami, FL 33145, USA

## Abstract

The production of ethanol has been considered as an alternative to replace part of the petroleum derivate. Brazil and the US are the leading producers, but more environmentally friendly alternatives are needed. Lignocellulose has an enormous potential but technology has to be still improve in order to economically produce ethanol. The present paper reviews the potential and problems of this technology and proposes the study of a group of microorganisms with the largest genetic pool, marine microorganism.

## Introduction

Countries all over the world are facing a rising demand for fuels, inflation, increase in energy prices, and issues on food and climate control. In 2010, the human population was estimated to be 6.8 billion and projected to reach 7.6 billion in 2020 (U.S. Census Bureau, Statistical Abstract of the United States 2011). At 3.000 Kcal per day, the world average per capita caloric food consumption (World Health Organization, 2003), the world in 2020 would demand the astonishing amount of 22.8 × 10^12^ Kcal per day, comparable to the energy of roughly 16 million barrels of oil equivalent (*boe*) per day.

The US is the largest petroleum consumer in the World. During 2010, consumed 19.1 million barrels per day, 49% of this was imported (US Energy Information Administration, 2011). Table 1 shows a comparison on energy consumption between Brazil, the US, China/India as the region most dense populated and the world. Interestingly, though the total vehicles in the US represent almost 24%, the US along consumes 42% of gasoline consumed in the world. These three country regions with 44% of the world population used 45% of the total energy and released 51% of the CO_2_ from the consumption of energy in the world (Table 1). 

**Table 1:** **Population, ethanol, energy consumption and CO**_**2 **_**emissions of Brazil, USA, China/India and in the World**

**Unit**	**Brazil**	**USA**	**China/India**	**World**
**Population (millions, 2010)**^**1**^	201.1 (2.9%)	310.2 (4.5%)	2,503.2 (36.5%)	6,853
**Total Vehicles (millions, 2010)**^**2**^	32.1 (3.2%)	239.8 (23.6%)	98.8 (9.7%)	1,015.3
**Vehicles per habitant**	0.159	0.773	0.0394	0.148
**Gasoline consumption, 2008 (thousand Barrels per day)**^**1**^	327.1 (1.5%)	8,989.2 (42.2%)	1,693.5 (7.9%)	21,323.1
**Fuel ethanol production, 2010 (thousand Barrels per day)**^**1**^	486.0 (32.0%)	867.4 (57.1%)	42.0 (2.7%)	1520
**Fuel ethanol consumption, 2010 (thousand Barrels per day)**^**1**^	381.9 (26.9%)	838.8 (59.1%)	38 (2.7%)	1,418.5
**Total Coal Consumption (Quadrillion Btu, 2010)**^**1**^	0.5 (0.3%)	20.8 (13.7%)	86.1 (56.8%)	151.5
**Total Petroleum Consumption ( Quadrillion Btu, 2010)**^**1**^	5.2	36.0	25.5	175.0
**Total Petroleum Consumption (Thousand Barrels Per Day, 2010)**^**1**^	2,560 (2.9%)	19,180.1 (22.0%)	12,507.9 (14.3%)	87,213
**Total Primary Energy Consumption (Quadrillion Btu, 2009)**^**1**^	10.3 (2.1%)	94.5 (19.6%)	111.9 (23.2%)	483.0
**Total Carbon Dioxide Emissions from the Consumption of Energy (Million Metric Tons, 2010)**^**1**^	453.9 (1.4%)	5,610.1 (17.7%)	10,016.6 (31.5%)	31,780.4

1. http://www.eia.gov/forecasts/ieo/index.cfm.

2. http://wardsauto.com/ar/world_vehicle_population_110815.

Worldwide, about 27% of primary energy is used for transportation, thus transportation fuels are promising targets to be replaced for a renewable source as well as for a reduction in greenhouse gas emissions (Antoni et al. 2007). At the end of the 19^th^ century, Henry Ford, and Nicholas Otto built engines that could run on ethanol. Then which better candidate than ethanol? Table 1 shows the amounts of vehicles in the world has already reached 1 billion units, and the US has by far the largest vehicle per habitant ratio in the world. The last century witnesses the rise, fall and the rebirth of ethanol (Solomon et al. 2007). Unfortunately, different factors played for the demand for alcohol to decrease and left only as an octane enhancer during and after the Great War. Despite health concerns the discovery of tetraethyl lead as antiknocking agent in the beginning of the 20’s, almost complete discontinued ethanol use in the car industry till the mid 70’s.

As a consequence of OPEC- Arab oil crisis of the 70’s, Brazil created in 1975 the National Ethanol Program (PROALCOOL) (Solomon et al. 2007). Under this program, tax and loan incentives were provided by the government to build production facilities. Brazilians incorporated the flexible fuel vehicles, more than any other country in the world, in this way Brazil became an energy independent nation largely to the adoption of legislation requiring ethanol use (Pilgrim, 2009).

In The US, ethanol industry was invigorated in the early 80’s when it became economical to produce ethanol by fermentation. By the 90’s MTBE (methyl tertiary- butyl ether), totally replaced lead additives as octane enhancer. Since 2000, the almost uncontrolled growth of supply and demand of ethanol, replacing MTBE (methyl tertiary- butyl ether), also alleviated some of the dependence to oil. In 2005, the renewable fuel standard (RFS) program was created under the Energy Policy Act (EPAct of 2005), and established the first renewable fuel volume mandate in the United States, this program required 7.5 billion gallons of renewable fuel to be blended into gasoline by 2012 (Environmental Protection Agency. 2005). From the environmental point of view, replacing MTBE and tetraethyl lead with ethanol as octane enhancer, reduced not only groundwater contamination but also harmful tailpipe emissions (e.g. for 2010, the production and use of more than 13 billion gallons of domestic ethanol reduced 21.9 million of tons of CO_2_e) (Renewable Fuels Association, 2011a).

The RFS was further expanded in 2007 under the Energy Independence and Security Act (EISA) of 2007 to include diesel, in addition to gasoline to increase the volume of renewable fuel required to be blended into transportation fuel from 9 billion gallons in 2008 to 36 billion gallons (bgy) by 2022. This constitutes a four-fold increase over current biofuel production and 25% of current motor fuel use. The mandate envisions eventual substitution of advanced cellulosic biofuels for currently produced corn based ethanol. But while achieving this goal, it doubles the use of corn based ethanol despite widespread concerns about increased food prices (Huffaker, 2010). A study carried out by the Department of Energy (DOE) and the USDA reported that over 1.3 billion dry tons per year of biomass potential, enough to produce biofuels to meet more than one-third of the current demand for transportation fuels, are available in the US (Perlack, et al. 2005). In Europe, the European Commission also plans to progressively substitute 20% of the conventional fossil fuels with alternative fuels in the transport sector by 2020 (Hahn- Hägerdal et al., 2006).

Corn used in the alcohol industry has led to record prices of over $8 per bushel in 2011, the demand has stimulated the planting of corn crops over the Corn Belt, and it is estimated that close to 90 million acres were planted in 2011 (USDA, 2011). Ethanol production from corn requires substantial amount of water (Mubako and Lant, 2008), part of it is consumed by evaporation from cooling towers and evaporators during distillation. For example, some ethanol plants in the US use from 3.5 to 6 gallons of water per gallon of ethanol produced (Institute for Agriculture and Trade Policy, 2006). Modern ethanol plants employ expensive water treatment technologies allowing unconsumed process water to be recycled within the plant, or discharged to freshwater sources (Huffaker, 2010). Mishra and Yeh (2010) estimated that the water requirement (gal/gal EtOH) to produce ethanol from corn was 2.72 and 4.11 for dry and wet milling respectively. Water demands of biorefineries, while modest compared with the irrigation demands of biofuel crops, could substantially affect local water users.

Few months ago, DOE published the goals for the biomass program for the next quinquennium. DOE encourage the production of biofuels nationwide and supports the EISA goal of 36 bgy of renewable transportation fuels by 2022 (US Department of Energy. 2011). The mission of the program is important to emphasize, “Develop and transform our renewable biomass resources into cost-competitive, high-performance biofuels, bioproducts, and biopower through targeted research, development, demonstration, and deployment supported through public and private partnership” (US Department of Energy. 2011). This program manages a diverse portfolio of technologies; one technology, biochemical conversion, is set forth to reduce the estimated processing cost for converting cellulosic feedstocks to ethanol from $1.85/ gallon in 2011 to $1.41 by 2012. In order to achieve this goal, the following two issues are critical: Lowering/stabilizing enzyme costs and the improvement of fermentative organisms and biochemical and conversion routes. The largest expected reduction in the cost of sugars will be obtained with biochemical conversion technology development in the areas of pretreatment, enzymes, and fermentation organisms. These steps represent $1.05/gallon from the total cost and are expected to be reduced to $0.63c/gallon by 2012.

### The technology: ethanol fermentation

**Figure 1:**
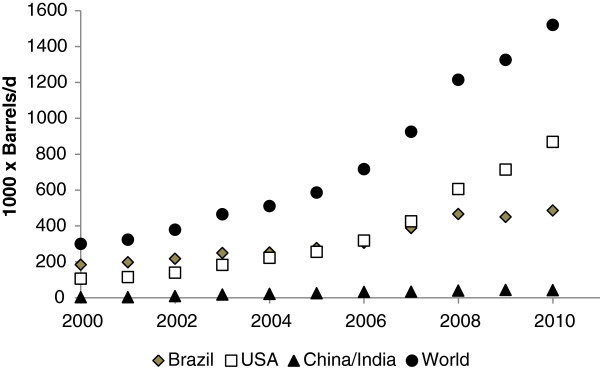
Ethanol production (thousands of barrels per day) in Brazil, US, China/India and in the world from 2000 to 2010.

Today most of the ethanol manufactured in the World is produced from sugar cane and corn from Brazil and the US, respectively, each country supplying almost equal volumes (Table 1, Figure 1). Current technology is based on sugar cane/corn starch conversion to ethanol utilizing yeast. In The US, corn grain ethanol plants produce from 10 to 15% ethanol, at rates ranging from 1.5 to 2.5 g/L/h (Haefele and Ross, 2009), reaching up to 20% ethanol when provided with very high substrate levels. Current ethanol production technology makes 2.7 to 2.8 gallons of fuel ethanol from a 56 lb. bushel of corn (Haefele and Ross, 2009). The production capacity of fuel alcohol in the US by 2011 was estimated to be 13.5 billion gallons with more than 204 operating plants in 29 states (Renewable Fuels Association, 2011b).

Ethanol production is carried out through a multistep process in a closed-loop biorefinery. The major process steps for a fermentation plant are as follows:

*Pretreatment*: Starchy feedstocks are broken down into component parts in preparation for hydrolysis. In a dry milling process, which is predominantly used in first-generation corn-based ethanol facilities, the feedstock is ground into flour and combined with water to make a mash. In wet-milling processes, feedstocks are combined with water and broken down into simpler parts using chemical processes, such as acid hydrolysis, fiber expansion, or alkaline wet oxidation.

*Hydrolysis*: Using enzymes, such as glucoamylase and alpha-amylase, the mash from the pretreatment phase is broken down further into component sugars.

*Fermentation:* The slurry from the hydrolysis phase undergoes an anaerobic process in which the sugars are converted into ethanol and carbon dioxide.

*Distillation*: The brew from the fermentation process is separated and concentrated into 95 percent pure alcohol, water, and other residues. Ethanol must be 95.6 percent by volume to be effective as a mobile fuel.

*Dehydration*: The resulting alcohol-water mixture is passed through molecular sieves to further absorb water and bring the ethanol mixture to 99.5 percent by volume. At this stage, denaturing agents are added to the ethanol mixture to render it unfit for consumption.

In addition, ethanol could also be produced from lignocellulosic biomass (grasses, agriculture residues, such as cobs, stalks, and leaves, wood wastes, fast growing trees, sugar wastes, citrus and rice wastes, the non-edible parts of plants and municipal wastes) (Lynd et al. 2005). Cellulosic ethanol is a new approach that may alleviate land use and related concerns. The obvious advantage of cellulosic ethanol is its dependence on abundant and diverse raw materials rather than traditional feedstocks, and because humans cannot digest cellulose, it does not compete with food production. Furthermore, exploiting the cellulose in corn plants or sugar cane, rather than the kernels, could double corn’s ethanol yield. Pimentel (2001) found that the corn- based ethanol yields net energy losses, but when the byproduct (e.g. DDGS, Distillers Dried Grains with Solubles, a livestock feed) of the distillates are included in the analysis, the ethanol production from corn yields 25% more energy than invested in its production (Hill et al., 2006).

### The problem

Terrestrial gross primary production ranges from 56 to 120 billion metric tons per year (Beer et al. 2010; Field et al. 1998). In nature, the two dominant simple sugars are D- xylose and D- glucose, 5 and 6 carbon sugars. These two sugars, in combination with other minor sugars, serve as building blocks for the production of more complex carbohydrates, such as lignocellulose, which is the structural component in the stem, trunk and leaves. Lignocellulose is mainly composed of cellulose and hemicellulose and lignin. Generally, lignocellulosic biomass contains 35 - 50% cellulose, 25 - 30% hemicelluloses and 20 - 25% lignin (Mabee et al., 2006; Girio et al. 2010).

Cellulose is the most abundant molecule in nature, it is a D- glucose polymer but its bond structure is the β -1, 4 linkages. Microbial degradation of cellulosic biomass is performed by mixtures of hydrolytic enzymes collectively known as cellulases. At least three enzymes have to cooperate: endoglucanase, exoglucanase, and β-glucosidase, which act in a synergistic manner in biomass degrading microbes. Some of the exoglucanases initiate their action from the end of cellulose chains and liberate cellobioses along cellulose chains (Murashima et al. 2002).

Some bacteria and fungi are capable of rapid and efficient degradation of cellulose (Lynd et al. 2002). The ability to degrade amorphous cellulose, soluble cellulose analogues, such as carboxymethyl cellulose, and hemicellulose-like substrates is relatively widespread. Cellulotic organisms and cellulases are extremely diverse. There are at least eleven cellulose families based on their similarities based on their amino acid sequence. Cellulase, contrasts from most enzymes because they degrade an insoluble substrate. Almost all enzymes that degrade insoluble substrates contain a substrate binding domain, which is usually joined to the catalytic domain (CBM) (Wilson. 2011).

Cellulases can also be susceptible to interference from various noncellulosic substances. Tejirian and Xu (2010) showed cellulases inhibition by ferrous/ferric ions. Their findings are supported by the fact that all microbial life cycles depend on redox chemistry, and many general microbial processes are of a redox nature. Microbes can be grouped as aerobes (reduces O_2_ to H_2_O), nitrate reducers or denitrifiers (reduces NO_3_^-^ to N_2_), Mn (IV) reducers (reduce MnO_2_ to Mn (II)), Fe (III) reducers (reduces Fe_2_O_3_ or FeO (OH) to Fe (II)), sulfate reducers (reduces SO_2_^2-^ or S^-^ to HS), or methanogens (reduce CO_2_ to CH_4_). They also found that strong iron ion chelators and polyethylene glycols could mitigate the inhibition.

Both anaerobic bacteria, as well as anaerobic fungi, are known to produce multienzyme complexes termed cellulosomes; in contrast, aerobic microorganisms produce their cellulase components as single enzymes. It has been suggested that the cellulosome provide anaerobic microorganisms with an advantage to degrade cellulose more efficiently, since cellulosomal cellulases degrade cellulose in a simultaneous manner rather than in a sequential manner (Murashima et al. 2002). Production of cellulosomes by anaerobic microorganisms is thought to be an evolutionary advantage which may counteract the low energy production by fermentation (Koukiekolo et al. 2005). Utilization of cellulosic biomass is more complex than using pure cellulose, because lignocellulose is a complex structure, in which cellulose and hemicellulose are encapsulated in lignin by hydrogen and covalent bonds, which makes the cellulose inaccessible for reaction with hydrolysis agents (Zinoviev et al. 2010). Hemicellulose, the second most abundant carbohydrate in nature and key component of cell wall structure, is made up of a mix of 5 and 6 carbon sugars, with the dominant sugar being xylose. In general, from 15 to 35% of the plant biomass is made up of hemicellulose (Girio et al. 2010), and its conversion into available fermentable sugars is the deciding element for the overall economization for the production of ethanol from lignocellulose (Chandel et al. 2011). Generally speaking none of the pentose sugars present in hemicellulose can be fermented to ethanol by the commercially ethanologenic available microbes (Antoni et al. 2007), as they lack the xylose utilization pathway consisting of xylose reductase and xylitol dehydrogenase. Many efforts have been made to construct such as strain by cloning pentose utilizing genes into ethanologenic microbes (Hahn- Hägerdal et al. 2007).

### Cellulosic alcohol technology

Traditionally, cellulosic ethanol focuses on producing sugars from biomass and fermenting those sugars into fuels. Himmel et al. (2007) summarized the natural factors believed to contribute to the recalcitrance of lignocellulosic feedstock to chemicals or enzymes. Normally, cultivation of cellulosic material with pure culture of cellulosic microorganisms results in a cellulose hydrolysis yield that is lower than 20% of the theoretical. As result cellulosic process design nearly always include a pretreatment step (Lynd et al. 2002). The lack of industrially suitable microbes for converting biomass into ethanol has been a major technical barrier. The characteristics required for an industrially appropriate ethanolic microorganism were summarized by Dien et al. (2003). Among them, ethanol yields higher than 90% of the theoretical, ethanol tolerance to higher than 40 g/L, an ethanol productivity of 1 g/L/h. However, no naturally occurring microorganisms can satisfy all of these features. One constraint is because native strains of ethanol-producing microorganisms, such as *S. cerevisiae*, *Pichia stipitis*, *Escherichia coli* and *Zymomonas mobilis*, cannot achieve effective co fermentation of glucose and xylose due to their intrinsic limitations. To solve this problem it is necessary to genetic modification to introduce pathways for xylose fermentation.

Current strategies to produce fuel ethanol from cellulose can be categorized depending on the process configurations, namely; separate hydrolysis and fermentation (SHF), which involves four discrete process steps. Simultaneous saccharification and fermentation (SSF) consolidates hydrolysis and fermentation of cellulose hydrolysis products into one process step, with cellulase production and fermentation of hemicellulose hydrolysis products occurring in two additional separate process steps. Simultaneous saccharification and co fermentation (SSCF) involves two process steps: cellulase production and a second step in which cellulose hydrolysis and fermentation of both cellulose and hemicellulose hydrolysis products occurs (Lee 1997; Lynd et al. 2002).

This technology has several problems; including the growth inhibition by harmful products formed during biomass hydrolysis, resulting in low alcohol concentration. These problems contribute to the high cost of lignocellulosic ethanol by increasing capital expenditure, reducing product yields, and increasing water volumes that must be handled as part of relatively dilute product streams. Similar to the traditional fermentation, the major process steps for cellulosic fermentation plant are as follows:

*Pretreatment*: Among of the chemical treatments used dilute acid at higher temperature has been widely used as it is highly active, inexpensive and easy to execute. In this step, biomass feedstock undergoes a process to break down the hemicellulose fraction of the feedstock into a mixture of soluble five-carbon sugars, such as xylose and arabinose, and soluble six-carbon sugars, such as mannose, galactose, and glucose, it also reduce cellulose crystallinity and increase the porosity of the materials (Mosier et al. 2005). This partial solubilization makes the remaining solid cellulose fraction more accessible for enzyme saccharification later in the process. A small portion of the cellulose is often converted to glucose in this step, as well as a portion of the lignin fraction may also be solubilized. Other chemical and physical methods such as ozonolysis, alkaline hydrolysis, oxidative delignification, solvent extraction, supercritical fluids, autohydrolysis, steam explosion and ammonia fiber expansion (AFEX) are also effective pretreatments (Girio et al. 2010). Pretreatment unavoidably yields fermentation inhibitors such as organic acids, furans, and phenols. In addition, pretreatment conditions must be tailor-made to the specific chemical and structural composition of the variable, sources of lignocellulosic biomass. Several detoxification methods such as neutralization, calcium hydroxide overliming, use of ion exchange resins, activated charcoal, and use of peroxidase or laccase have shown promising results (Chandel et al. 2011). There is not ideal pretreatment, the most appropriated treatment depend on the recalcitrance of the type of material (Girio et al. 2010).

*Conditioning:* In some process configurations, the pretreated material goes through a hydrolysate conditioning and/or neutralization process which removes undesirable toxic byproducts formed in the pretreatment process and adjusts the pH of the reactant.

*Hydrolysis/Saccharification*: The pretreated material with the remaining solid carbohydrate fraction, primarily cellulose and hemicellulose, is saccharified, releasing glucose and pentose sugars. This can be done with enzymes such as cellulases and hemicellulases. Depending on the process design, enzymatic hydrolysis requires from several hours to several days, after which the mixture of sugars and any unreacted cellulose is transferred to the fermented.

*Biological Processing*: Currently a fermentation step, an inoculum of a fermenting organism is added and fermentation of all sugars to ethanol is carried out while continuing to utilize the enzymes for further glucose production from any remaining solid cellulose. After a few days of fermentation and continued saccharification, nearly all of the sugars are converted to ethanol. The distillation and dehydration steps are similar to the traditional ethanol production describes above. It is important to point out that without the use of the pentose sugars release from the hemicellulose fraction, the ethanol production will not be able to compete with the ethanol from the grain counterpart.

The discovery or engineering of microorganisms with the ability to convert more efficiently the components of lignocellulosic biomass into sugars has a high priority (Yang et al. 2009). This approach, and perhaps the most promising, is consolidated bioprocessing (CBP), in which cellulase production, substrate hydrolysis, and fermentation are accomplished in a single process step by cellulolytic microorganisms (Lynd et al. 2005). Microorganisms required for CBP are not commercially available; although many research labs and companies are extensively searching. One characteristic to further differentiate CBP with traditional methods is that there is an apparent enzyme- microbe- synergy that results in better cellulase production and hence better ethanol yield.

### Noisy microorganisms

Bacteria the oldest organisms on earth first appeared from the Ocean more than 3 billion (Dagan et al. 2010). Eukaryotes appeared about 2 billion years later, first as single heterotrophic cells followed as eukaryotic algae (Dagan et al. 2010). Among the Eukaryotes, fungi play an important role in the mineralization of organic matter in all environments. It is appropriate to note that the domestication of *Saccharomyces cerevisiae* can be considered a significant event in human history (reviewed by Mortimer, 2000; Johnson and Echavarri- Erasun 2011). Today, yeasts are the major producer of biotechnology products worldwide, exceeding production, in capacity and economic revenues, to any other group of industrial microorganisms. Traditional industrial uses of yeasts include in the fermentations of beers, cider, wines, sake, distilled spirits, bakery products, cheese and sausages. Other established industrial processes that involve yeasts are the production of fuel ethanol, single cell protein, feeds and fodder, industrial enzymes, and small molecular weight metabolites. Yeasts, especially *S. cerevisiae*, are increasingly being used as hosts for expression of protein biocatalysts and multi-enzyme pathways for the synthesis of fine chemicals and small molecular weight compounds of medicinal and nutritional importance. It is also the principal model eukaryotic organism utilized for fundamental research.

Microorganisms also exhibit remarkable social behavior, which amusingly some researchers have proposed are similar to those performed by insects, vertebrates and humans (Diggle et al. 2007). This conduct result in the ability to detect and respond to changes in the environment for their survival, and as a result, a variety of mechanisms have developed by which organisms sense their environment and respond to signals that they detect (Zhou et al. 2007). All the above lead to the use of communication, and in microbes it is done by secreting signaling molecules called auto inducers, a process known as quorum sensing (QS) (Lerat and Moran, 2004; Waters and Bassler, 2005; Gera and Srivastava, 2006). Quorum sensing was first designated by Fuqua et al. (1994) to describe the phenomenon whereby the accumulation of signaling molecules allows a single cell to sense the number of bacteria and therefore the population as a whole can make a coordinated response. Zhou et al. (2007) suggested that a common “noisy” signaling molecule could initiative a collective of uncoupled genetic oscillators which will developed an “on- off” gene expression switch that is sensitive to environmental control and yet highly robust to intracellular molecular noise.

In addition, QS is widely recognized as an efficient mechanism to regulate expression of specific genes responsible for the successful establishment of symbiotic, pathogenic, or commensal relationships with eukaryotic hosts, including motility, exopolysaccharide production, biofilm formation, toxin production, mating, virulence and production of metabolites (Gera and Srivastava, 2006; Gonzalez and Keshavan, 2006). This noisy communication may take different forms, such as the acyl -homoserine lactones (AHL) used for Gram-negative bacteria, peptides used in Gram positive bacteria. Another class of molecules includes metabolic byproducts such as acetate or lactate that are typically not considered as cell to cell communication molecules but, can be used effectively for such purposes (Bulter et al. 2004; Gerchman and Weiss, 2004). In this connection, sub lethal allelopathy could also be particularly effective in shaping community structure and function by altering growth and loss rates (Strom, 2008).

The fungal pathogen *Candida albicans* was the first eukaryotic microorganism shown to exhibit QS (Mohammed et al. 2006). Tyrosol and farnesol are QS molecules produced by *C. albicans* which accelerate and block, respectively, the morphological transition from yeasts to hyphae. It appears; therefore, that morphogenesis in *C. albicans* is under complex positive and negative control by the actions of tyrosol and farnesol, respectively (Mohammed et al. 2006; Nickerson et al. 2006).

As such, the ability to block or promote these systems provides a powerful tool to solve many problems and enhance productivity in microbes used in industry. For instance, Butanediol fermentation in two *Serratia* species has been shown to be dependent of quorum sensing (Van Houdt et al. 2006). Iida et al. (2008) demonstrated a relationship between quorum sensing and oxidative fermentation in acetic acid bacteria. They also postulated that manipulation of the quorum-sensing system is expected to be applicable to the industrial production of not only acid production but also various other materials.

In *Vibrio cholerae*, QS and 3', 5’- cyclic diguanylic acid (c-di-GMP), reciprocally control biofilm formation (Waters et al. 2008). The biofilm is the preferred lifestyle in the microbial world as it enhances growth and survival by improving the availability to nutrients and shelter from predators and antimicrobials, thus providing a substantial survival advantage to aquatic organisms such as *Vibrio* species (Yildiz and Visick, 2009). Observations have conclusively shown that biofilm bacteria (attached) predominate, numerically and metabolically, in virtually all ecosystems. A number of phenotypic characteristics of *Chromobacterium violaceum* ATCC 31532, including production of the purple pigment violacein, hydrogen cyanide, antibiotics, exoproteases and chitinolytic enzymes are known to be regulated by the endogenous AHL*N*-hexanoyl-L-homoserine lactone (HHL) (Chernin et al. 1998). DeAngelis et al. (2008) has postulated that the complex process of rhizosphere nitrogen mineralization could also be controlled by QS activity. In addition to their predominant role of QS in nutrient cycling in soil, QS play a similar key role in wastewater treatment systems. Thompson et al. (2006) showed that in general nutrient limitation may stimulate the production of QS and as a result biofilm formation.

### Fuel forces

Microorganisms are usually grouped into those relying solely on harvesting light, or those relying on the assimilation of organic or inorganic compounds to meet their requirements for energy. Likewise, most biogeochemical studies group microorganisms into photo autotrophs like micro algae and cyanobacteria and heterotrophs, like most heterotrophic bacteria. Although this simple classification cannot described the metabolic diversity regarding the role of bacteria in the carbon cycle (Eiler 2006), heterotrophic bacteria predominate the photic zone of Oceans. These bacteria utilize dissolved organic matter excreted mainly by phytoplankton and its dependence is such that there is a close correlation between bacterial and primary production (Azam et al. 1983). Cole et al. (1988) estimated that bacterial production in the water column represented 20% of planktonic primary production.

In contrast to the photic zone of Oceans, the major source of organic matter in swamps, sea grass meadows and estuaries is detritus derived from vascular plants. The bulk of vascular plant detritus is composed of the structural polymers commonly referred to as lignocellulose. Usually less than 10% of litter material produced in mangrove forests is consumed alive (Bosire et al. 2005), the remnants become part of the detritus food chain. Wilson (1985) found that 80% of the decomposition from *Spartina alterniflora* is largely decomposed *in situ*, and after 1 year only 10 to 40% of the initial weight of plant litter still remains. Regardless of what initially thought both lignin and lignified plant tissues can also be degraded in the anaerobic sediments (Benner et al. 1984). The degradation of organic matter in mangrove sediments is mediated by both aerobic and anaerobic microbial processes using a variety of electron acceptors (Kristensen et al. 2008).

In this connection degradation of the complex component of plant material such as mangrove tissue required very resourceful microorganism that not only degrade mangrove constituents’ compounds, such as carbohydrates, amino acids, lignin derived phenols, fatty acids, triterpenoids and n-alkanes, but also can use or tolerate high concentrations of tannins. Tannins are major constituents of vascular plants, along with lignocellulose, and sometimes comprise more than 20% of the dry weight of plant materials (Maie et al. 2008). Green leaves of *Rhizophora mangle* may contain more than 6% of tannins (Kristensen et al. 2008).

### Nutrient cycling

Diatoms, which are responsible for approximately 20% of annual primary production and support the most biologically productive regions of the ocean, are known to be chitin producers. The deposition of chitin at diatom girdle bands has the potential to facilitate diatom- microbe interactions, making an attractive source of nutrients for microbes. In this way, chitin functions as an enormous reservoir of organic carbon and nitrogen in the environment (Blokesch and Schoolnik, 2007; Durkin et al. 2009). Chitin, a polymer of N- acetylglucosamine is the most abundant polymer in the ocean and the second most abundant polymer on earth, surpassed only by cellulose (Durkin et al. 2009). In the aquatic biosphere alone, more than 100 billion metric tons of chitin is produced annually. This huge amount of insoluble material is recycled mainly by chitinolytic bacteria, including members of the genera *Vibrio*, *Aeromonas*, *Alteromonas, Enterobacter, Pseudomonas, Serratia*, *Ewingella* and *Chromobacterium* (Chernin et al. 1998; Meibom et al. 2004). Much of the chitin found in oceans is rapidly degraded while in suspension, but some is incorporated into sediments. Anaerobic degradation and utilization of chitin in ocean sediments, similarly to the anaerobic degradation of cellulose in terrestrial environments, is thought to be coupled to processes such as methanogenesis or sulfate reduction via interspecies hydrogen transfer (Reguera and Leschine, 2001). Chitin serves as a nutrient for *V. cholerae* and it induces natural transformation, a process by which it acquires new genes from other microbes in the same habitat (Blokesch and Schoolnik, 2007). The *V. cholerae* link with chitin is an extensively documented phenomenon and best examples of a successful bacteria-substrate interaction (Pruzzo et al. 2008). Chitin utilization is important at the ecosystem level by contributing to both C and N recycling. This polymer is one of the most abundant and important sources of nutrients and energy in the marine environment. It is distributed throughout all kingdoms, as it is a crucial component of the cell walls of moulds, yeasts, fungi and certain green algae, and is a major component of the cuticles and exoskeletons of worms, mollusks and arthropods. Attached bacteria metabolize chitin more efficiently than free living bacteria, thus increasing the rate of chitin mineralization in the natural environment.

### Cellulose degraders

Temperature and pH are major environmental variables that regulate rates of lignocellulose mineralization to CO_2_, as well as the C/N ratio and lignin content (Bridgham and Richardson, 2003). The production of microbial biomass at the expense of lignocellulose is a major pathway of carbon and energy flow in these ecosystems (Benner et al. 1984; Benner and Vaun McArthur, 1988; Mann, 1988). Important metabolic differences between fungi and bacteria suggest that the contributions to plant decomposition in the marine environment by these microorganisms will vary with both physical and chemical aspects of the substrate. High a high C/N ratio is believed to stimulate fungi, whereas increasing levels of N would increase bacterial growth. On the other hand, Pascoal and Cassio (2004), found that leaf decomposition tends to be faster at nutrient polluted sites, with nitrogen and/or phosphorus reported to stimulate both fungal and bacterial activities. Fungi, in particular aquatic hyphomycetes, recognized as dominant players in microbial decomposition of leaf litter in streams, whereas bacteria are thought to increase their importance only after leaf material has been partially broken down. It seems more likely that the natural chemical composition of the substrates, including the solubility and availability of various compounds as well as environmental factors such as temperature, concentration of dissolved nutrients, and pH contributes more to the explanation (Rousk and Baath, 2007). Decades of marine mycology have clearly demonstrated that marine fungi differ from their terrestrial and freshwater counterparts, both in their taxonomy, morphology and adaptation to the aquatic environment (Jones, 2000). Some of these fungi however, may be present in either aquatic terrestrial habitat. Although, no single factor can account for the observed diversity, again salinity and temperature are the major factors affecting the variety of marine fungi.

Statzell Tallman et al. (2008) found that fungi and fungal like organisms play a major role in the mangrove ecosystems, converting lignin and cellulose and its leachates, into microbial protein. Newell et al. (1987) and Newell and Fell (1997) suggested that oomycotes, are major mycelial decomposers of submerged leaves. The oomycotes are mycelial protoctists that have swimming propagules (zoospores) resembling planktonic heterotrophic flagellates. The degradation mechanisms of wood by terrestrial fungi are well known and it is assumed that similar mechanisms exist in marine fungi. Bucher et al. (2004), demonstrates the ability of certain marine ascomycetes to solubilize lignin from wood is equivalent to known terrestrial white- rot basidiomycetes.

Sea grass meadows are, productive ecosystems with an average standing stock sea grass dry weight of 460 g per m^2^ (Duarte and Chiscano, 1999). During the past few years, more attention has been given to the role of bacteria in the decomposition of lignin and lignocellulosic plant materials (Kerr et al. 1983). Sea grass roots are colonized by anaerobic bacteria, which not only contribute to the vitality of sea grasses but also to the biogeochemistry of the surrounding sediment (Kusel et al. 1999; Kusel et al. 2001; Nielsen et al. 1999). Kusel et al. (2001) isolated an ethanol producing anaerobic Gram Positive bacterium, from the root of the sea grass *Halodule wrightii*, which metabolizes certain substrates via the acetyl-CoA pathway, these bacteria could also tolerate and consume limited amounts of O_2_, which enhance the production of ethanol, lactate, and H_2_. It was suggested the ability to cope with limited amounts of O_2_ might contribute to its survival in a habitat subject to daily gradients of O_2_. In addition, when the transport of O_2_ to the sea grass roots is not enough to meet the demand for aerobic respiration, then sea grasses, may switch to a fermentation pathway releasing ethanol for short time periods (Smith et al. 1988).

Many microorganisms can attack cellulose, but few taxa can completely degrade it. In terrestrial environments, cellulose tends to be highly lignified and more difficult to degrade; both fungi and Actinomycetes can obtain access to cellulose in woody tissue due to their hyphal growth form. In contrast, little is known about the contributions of different groups of microorganisms to cellulose degradation in aquatic ecosystems. Bacteria appear to be the major degraders of lignocellulose in many aquatic environments (Benner et al. 1986). Gonzalez et al. (1997) found that while both bacteria and fungi can be involved in the degradation of lignin, bacteria are probably responsible for the utilization of the most refractory components. Moran and Hodson (1989) found that Bacteria are capable of using both the soluble fraction of the vascular plant, and the highly refractory lignocellulosic fraction, which is deposited as particulate detritus. They also found that degradation of plant material is predictable as its rate of degradation dependent on nitrogen, lignin content, and the C/N ratio. Both sea grasses and mangrove are the major contributors of lignocellulose in coastal marine ecosystems, and although contrary reports fungi play an important role in lignocellulose degradation in these ecosystems (Raghukumar et al. 1999).

In aerobic systems, cellulose is commonly degraded into H_2_O and CO_2_ while in anaerobic systems both CH_4_ and H_2_ are also produced. Cellulolytic species are found within the phyla Thermotogae, Proteobacteria, Actinobacteria, Spirochaetes, Firmicutes, Fibrobacteres and Bacteroids. Of these, approximately 80% of the isolated cellulolytic bacteria are found within phyla Firmicutes and Actinobacteria. The majority of the gram-positive cellulolytic bacteria is found within Firmicutes and belongs to the class Clostridia and the genus *Clostridium* (Carere et al. 2008). Among the cellulolytic bacteria, Clostridia have been best studied and characterized. In marine environments, sulfate is plentiful and sulfate reducing bacteria out compete methanogens for H_2_S (Leschine, 1995).

The microbial degradation of cellulose and hemicellulose is not as well characterized in the oceans as it is in terrestrial systems (Taylor et al. 2006). More than a few marine cellulosic bacterial have been isolated. For instances, *Saccharophagus degradans* a pleomorphic, gram-negative, aerobic, motile γ-Proteobacteria isolated from decaying salt marsh. *S. degradans* can utilize at least 10 distinct complex polysaccharides from diverse algal, plant and invertebrate sources (Suvorov et al. 2011; Taylor et al. 2006).

Several cellulolytic anaerobes have been isolated from the marine environment (Lynd et al. 2002). *Clostridium cellulovorans*, an anaerobic bacterium, degrades native substrates efficiently (Koukiekolo et al. 2005). *Micromonosporas* are a group of Actinomycetes that are usually present in large numbers in soil but are also well adapted to seawater (de Menezes et al. 2008; Mincer et al. 2002; Moran and Hodson, 1989). These bacteria are primarily saprophytic and are best known from sediments where they contribute significantly to the turnover of complex biopolymers, such as lignocellulose, hemicellulose, pectin, keratin, and chitin (Mincer et al. 2002). *Anaerocellum thermophilum*, an anaerobic thermophilic bacterium that grows optimally at 75°C, efficiently utilizes various types of untreated plant biomass, such as; hardwoods such as poplar, low-lignin grasses Bermuda grasses, and high-lignin grasses such as switch grass as well as crystalline cellulose and xylan (Ranatunga et al. 1997).

Reguera and Leschine (2001), isolated a facultative aerobic bacteria, identified as *Cellulomonas uda.* This bacteria, could utilized chitin as a source of nitrogen for the degradation of cellulose. It should be taken into account that environments where cellulose accumulates are frequently deficient in nitrogen which may limit plant litter decomposition. In natural environments, the ability to use chitin as a nitrogen source may confer on cellulolytic microorganisms, a selective advantage over other cellulolytic microbes. The genus *Cellulomonas* comprises facultative aerobic bacteria that have been traditionally characterized by their ability to degrade cellulose. All members of the genus *Cellulomonas* that were examined in this study also degraded chitin, both aerobically and anaerobically, suggesting that the ability to degrade chitin might be widespread among cellulolytic bacteria from terrestrial environments.

## Conclusions and recommendations

The ocean is the mother of life and it is believed that the most primitive forms of life originated from a “primordial soup”. It contains a massive variety of marine organisms that are diverse in their physiology and adaptations. Hence, oceans may be considered to be rich in organic compounds favorable for the evolution and growth of life in general. Most of the Earth’s microbial diversity is found in the ocean, which ultimately directs an enormous number of bioactive substances (Bhatnagar and Kim, 2010). The oceans contain environments that resemble those that first supported life on earth (Bowler et al. 2009), stress, competition for space and nutrients in the marine environment is a powerful selective force, which has led to endless evolution. Salinity, pressure, temperature and special lighting conditions, contributed to the significant differences between the enzymes generated by marine microorganisms and homologous enzymes from terrestrial microorganisms. Thus constant evolving microbes became the biogeochemical engineers of life on earth (Bowler et al., 2009; Falkowski et al. 2008; Fuhrman, 2009; Zhang and Kim, 2010). There is not organism on earth that could match bacteria importance on number, diversity and its adaptation capability. For instances, the microbiome of the human gut flora comprised of 500 to 1000 bacterial species with two to four million genes and 10^13^ bacterial cells, exceeding by 100 and 10 fold the genes of the human genome and the total number of body cells respectively (Sears, 2005). Earth’s ocean is estimated to enclose 10^29^ bacteria, a number superior than the estimated 10^21^ stars in the universe, and its total mass of bacteria exceeds the combined mass of zooplankton and fishes (Pomeroy et al. 2007). In addition, phages which drive the dynamic of marine microbial food web are known to carry and transfer a variety of host genes, as serve as gene reservoirs that change the ecological niche of the reservoir (Rohwer and Vega Thurber, 2009). Production of fuel ethanol from lignocellulosic biomass remains challenging, with many opportunities for improvement. The isolation, characterization and culture of potential microbial strains from the marine environment remain intact. For instances, bacterial communities associated with the wood-feeding molluscs, have been shown to have remarkable cellulosic properties (Zbinden et al. 2010). The gram-negative bacterium *Teredinibacter turnerae* isolated from the mangrove shipworm *Neoteredo reynei* is a symbiont that can use cellulose as the sole carbon source and fix dinitrogen under micro-aerobic conditions. *T. turnerae* is the only known bivalve-gill endosymbiont that can be cultured, and has been shown to present a potential for biotechnological application. (Distel et al. 2002; Trindade-Silva et al. 2009). *T.turnerae* are also capable of using xylan, pectin, CMC, cellobiose and a wide variety of sugars and organic acids. Interestingly, these shipworms isolates are unique in the capacity of growing in pure cultures, and with cellulose and di nitrogen is a rare combination.

Similar to the relationship chitin-*V.cholerae*, close associations with cellulose has not been explored. For instances, Cellulose is common in appendicularians and thecate dinoflagellates (Kimura et al. 2001; Kwok and Wong 2003), therefore a relationship or bacteria associated to the degradation of this polysaccharide could exist.

It is clear than tougher microorganisms are needed with higher rates of conversion and yield to allow process simplification through consolidating process steps. Microorganisms from extremes environments such as thermophilic and acidic vents or sea grass sediments where both oxygen fluctuations and ethanol release from the roots provide the correct niche to find the right strains (cellulose degrader, ethanol producing and tolerant strains), the use of blockers or inducers (QS) as a enhancing tool could also result in the isolation of right strains needed by the industry. In addition, phages are known to carry a variety of host genes, not only in a negative effect but as it has been shown it can also augment the metabolism, immunity and distribution of the host in many unexpected ways (Rohwer and Vega Thurber, 2009), it would not be wrong to suggest that the marine environment carry the right soup to obtain the desirable result.

## Competing interests

The author declare that he have no competing interests.
